# The pharmacokinetic study on the interaction between nobiletin and anemarsaponin BII *in vivo* and *in vitro*

**DOI:** 10.1080/13880209.2021.1990355

**Published:** 2021-11-02

**Authors:** Jie Zhang, Meiling Fan, Xia Yu, Bin Zhang

**Affiliations:** a Department of Medicinal Medicine, The Second Hospital of Shandong University, Jinan, Shandong, China; b Department of Medicine, Qingdao Municipal Hospital (East Campus), Qingdao, Shandong, China; c Department of Anesthesia and Perioperative Medicine, Dongying Hospital of Traditional Chinese Medicine, Dongying, Shandong, China

**Keywords:** Paediatric, traditional Chinese medicine, herb–herb interaction, CYP3A4, P-*gp*

## Abstract

**Context:**

The interaction between nobiletin and anemarsaponin BII could affect the pharmacological activity of these two drugs during their combination.

**Objective:**

The co-administration of nobiletin and anemarsaponin BII was investigated to explore the interaction and the potential mechanism.

**Materials and methods:**

Male Sprague-Dawley rats were only orally administrated with 50 mg/kg nobiletin as the control and another six rats were pre-treated with 100 mg/kg anemarsaponin BII for 7 d followed by the administration of nobiletin. The transport and metabolic stability of nobiletin were evaluated *in vitro*, and the effect of anemarsaponin BII on the activity of CYP3A4 was also assessed to explore the potential mechanism underlying the interaction.

**Results:**

The increasing C_max_ (2309.67 ± 68.06 μg/L vs. 1767.67 ± 68.86 μg/L), AUC (28.84 ± 1.34 mg/L × h vs. 19.57 ± 2.76 mg/L × h), prolonged t_1/2_ (9.80 ± 2.33 h vs. 6.24 ± 1.53 h), and decreased clearance rate (1.46 ± 0.26 vs. 2.42 ± 0.40) of nobilein was observed in rats. Anemarsaponin BII significantly enhanced the metabolic stability of nobiletin in rat liver microsomes (half-life increased from 31.56 min to 39.44 min) and suppressed the transport of nobiletin in Caco-2 cells (efflux rate decreased from 1.57 ± 0.04 to 1.30 ± 0.03). The inhibitory effect of anemarsaponin BII on CYP3A4 was also found with an IC_50_ value of 10.23 μM.

**Discussion and conclusions:**

The interaction between anemarsaponin BII and nobiletin was induced by the inhibition of CYP3A4, which should draw special attention in their clinical co-administration.

## Introduction

Fever and cough are common paediatric diseases in the clinic, and traditional Chinese medicine is always considered as the main therapeutic strategy in China (Shih et al. 2016; Wang C et al. 2019). The combination of various herbs is the main trait of traditional Chinese medicine that could improve the curative effect and treat the complications. Due to similar indications, *Anemarrhena asphodeloides* Bunge (Asparagaceae) and *Citrus reticulata* Blanco (Rutaceae) might be used in the same prescription (Wang Y et al. 2014; Liu et al. 2016). Nobiletin is a kind of polymethoxyflavonoid extracted from the leaves or stem of *C. reticulata*. In previous studies, the pharmacological activities of nobiletin have been widely reported including anticancer, anti-inflammation, antioxidant, and neuroprotection (Kazak et al. 2021; Rong et al. 2021; Wang JG et al. 2021). Anemarsaponin BII is the major active ingredient of *A. asphodeloides* responsible for the pharmacological effects, such as anti-inflammatory, immune-stimulating, and treating paediatric fever, cough, and allergies (Kim et al. 2009; Zhao X et al. 2016).

Additionally, both anemarsaponin BII and nobiletin have been reported to possess the protective effect against epilepsy, which makes them easier to be co-administrated (Wang Y et al. 2014; Yang et al. 2018). Therefore, the investigation on the interaction between anemarsaponin BII and nobiletin is necessary for their clinical application, especially for their co-administration. The drug-drug interaction between different drugs has been reported widely in previous studies, which has been considered as a vital factor that should be included in the clinical prescription of drugs (Zhao Z et al. 2016). For example, peimine and paeoniflorin are the major extractions of herbs used for cough in paediatrics, and the co-administration of these two compounds results in the inhibition of the metabolism of paeoniflorin (Chen et al. 2021). The interaction between nobiletin and anemarsaponin BII was clear, which is of great importance for the clinical combination of *C. reticulata*. and *A. asphodeloides*.

This study investigated the co-administration of nobiletin and anemarsaponin BII *in vivo* and *in vitro* to disclose the potential interaction between nobiletin and anemarsaponin BII and provide guidance for the clinical prescription of *C. reticulata*. and *A. asphodeloides*.

## Materials and methods

### Animals

Male Sprague-Dawley rats (weighing 220 ± 20 g) obtained from the Shanghai Experimental Animal Centre of the Chinese Academy of Science (Shanghai, China) were used in this study. All rats were housed in a clean and well-ventilated animal room with standard conditions and free access to diet. Healthy rats were selected and fasted overnight before the experiments. This study was approved by the Animal Care and Use Ethics Committee of The Second Hospital of Shandong University.

### Grouping and pharmacokinetic study

The experimental animals were divided into four groups with six rats in each group. The treatment methods and the dosage of two compounds were applied according to previous reports (Wang H et al. 2020; Wang M et al. 2020). The rats in the nobiletin group were orally administrated with 50 mg/kg nobiletin alone. The rats in the nobiletin + anemarsaponin BII group were pre-treated with 100 mg/kg/day anemarsaponin BII for 7 d followed by the administration of 50 mg/kg nobiletin. The anemarsaponin BII group was orally administrated with 100 mg/kg anemarsaponin BII, and the anemarsaponin BII + nobiletin were pre-treated with 50 mg/kg/day nobiletin for 7 d followed by 100 mg/kg anemarsaponin BII. The blood samples were collected after 0, 0.083, 0.33, 0.5, 1, 2, 4, 6, 8, 10, 12, and 24 h of the administration of nobiletin and centrifuged to obtain plasma samples. The plasma concentration of nobiletin was analyzed by HPLC to evaluate the change of nobiletin pharmacokinetics.

### Transport study in Caco-2 cell

Before the experiments, the Caco-2 cells were incubated in DMEM high glucose medium (Life Technologies, USA) with 15% FBS (Life Technologies, USA) and 1% penicillin and streptomycin (Sigma, USA) at 37 °C with 5% CO_2_. The cultured cells were seeded into the Transwell chambers with a pore size of 0.4 μm (Corning, USA) and incubated for 21 d to obtained Caco-2 cell monolayers. After the incubation, the cell monolayers were washed with HBSS twice and incubated at 37 °C for 20 min. Then, the cell monolayers were mixed with nobiletin in a fresh medium and incubated for 30, 60, 90, and 120 min. The transport of nobiletin was evaluated from the apical side to the basolateral side and in the opposite direction. The activity of *P-gp* was estimated with the employment of verapamil (typical inhibitor) and rhodamine (typical substrate).

The values of P_app_ and efflux ratio were calculated to assess the transport of nobiletin with the following equations:

$$\begin{array}{c} P_{\text{app}}=\;(△Q/△t)\;\times \;\left[1/\left(A\;\times {\;C}_{0}\right)\right]\\ \text{Efflux}\;\text{ratio}=P_{\text{appBA}}/P_{\text{appAB}} \end{array}$$

where P_appBA_ is the apparent permeability coefficient from the basolateral side to the apical side and P_appAB_ is the apparent permeability coefficient from the apical side to the basolateral side (cm/s). △*Q*/△*t* is the rat of nobiletin that appears in the bottom chamber. *A* is the surface area of the cell monolayers and *C*_0_ is the initial concentration of nobiletin in the top chamber.

### Metabolic stability evaluation in rat liver microsomes

The *in vitro* experiments were conducted to evaluate the metabolic stability of nobiletin. The incubation included an NADPH-generating system (10 mM G-6-P, 1 mM NADP^+^, 4 mM MgCl_2_, and 1 unit/mL G-6-PDH), 30 μL rat liver microsomes, 12 μL nobiletin, and 1113 μL PBS buffer. There was a 5 min preincubation before the reaction was initiated by a 45 μL NADPH-generating system. After incubating for 0, 1, 3, 5, 15, 30, and 60 min, 100 μL mixture was collected and centrifuged to evaluate the concentration of nobiletin.

The metabolic stability of nobiletin was evaluated by the corresponding parameters, including the half-life (*t*_1/2_) and intrinsic clearance rate calculated by the following equations:

$$\begin{array}{c} t_{1/2}=\;0.693/k;\\ V\;\left(\mu L/\text{mg}\right)=\text{volume}\;\text{of}\;\text{incubation}\;\left(\mu L\right)/\text{protein}\;\text{in}\;\text{the}\;\text{incubation}\;\left(\text{mg}\right);\\ \text{Intrinsic}\;\text{clearance}\;\left(\mu L/\text{min}/\text{mg}\;\text{protein}\right)=\;v\;\times \;0.693/t_{1/2} \end{array}$$

### CYP3A4 activity assessment in rat liver microsomes

The incubation system was similar to the above system with testosterone, a typical substrate of CYP3A4, and anemarsaponin BII at concentrations of 0, 2.5, 5, 10, 25, 50, and 100 μM. The reaction was initiated by adding the NADPH generating system after a 5 min preincubation and terminated by adding ice-cold acetonitrile. The mixture was collected and centrifugated for the analysis of CYP3A4 activity.

### Statistical analysis

All data were expressed as mean value ± SD obtained from triplicate experiments. The corresponding pharmacokinetic parameters of nobiletin, including the area under the plasma concentration-time curve (AUC), maximal plasma concentration (*C*_max_), the time reached *C*_max_, the half-life (*t*_1/2_), and the total clearance (CL/F) were obtained with the help of the DAS 3.0 pharmacokinetic software (Chinese Pharmacological Association, China). The difference between the two groups was assessed by the Student’s *t*-test with SPSS 20.0 (Chicago). *p* < 0.05 was considered to be statistically significant.

## Results

### The pharmacokinetic interaction between anemarsaponin BII and nobiletin in rats

**Figure 1.**
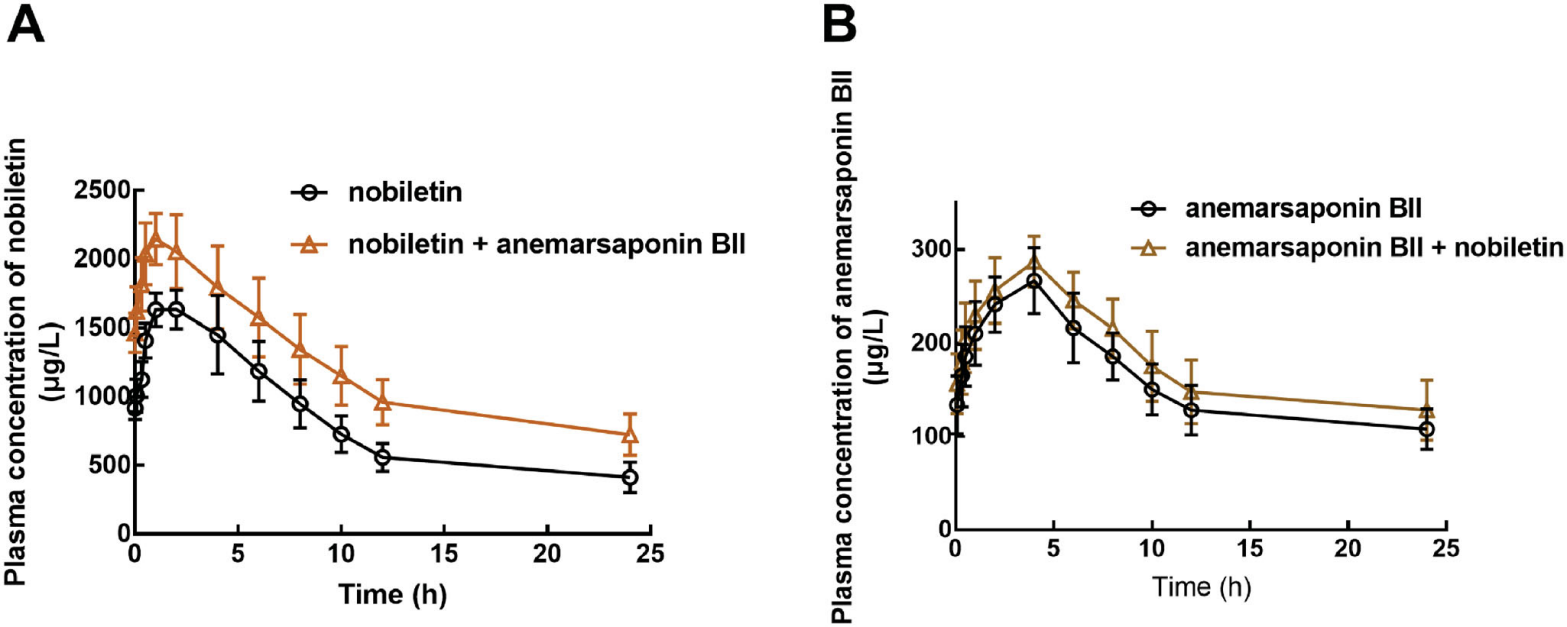
The pharmacokinetic profile of nobiletin (A) and anemarsaponin BII (B) with or without co-administration.

The plasma concentration-time curve of nobiletin and anemarsaponin BII is shown in Figure 1. It was observed that the pre-treatment of anemarsaponin BII significantly changed the pharmacokinetic profile of nobiletin (Figure 1(A)). Specifically, the AUC of nobiletin was 19.57 ± 2.76 mg/L × h with the *C*_max_ of 1767.67 ± 68.86 μg/L and the *T*_max_ of 1.83 ± 1.17 h (Table 1). In the presence of anemarsaponin BII, the AUC of nobiletin increased to 28.84 ± 1.34 mg/L × h and the increasing *C*_max_ of 2309.67 ± 68.06 μg/L. The t_1/2_ was prolonged from 6.24 ± 1.53 h to 9.80 ± 2.33 h (Table 1). Additionally, the clearance of nobiletin was found to decrease from 2.42 ± 0.40 L/h/kg to 1.46 ± 0.26 L/h/kg (Table 1). While the pharmacokinetic profile of anemarsaponin BII was not affected by the co-administration of nobiletin (Figure 1(B)). The corresponding parameters also showed no significant difference (Table 1).

**Table 1.** Corresponding pharmacokinetic parameters of nobiletin (50 mg/kg) and anemarsaponin BII (100 mg/kg) with different pre-treatment.

	Nobiletin	Nobiletin + anemarsaponin BII	Anemarsaponin BII	Anemarsaponin BII + nobiletin
AUC _(0–_*_t_*_)_ (mg/L*h)	19.57 ± 2.76	28.84 ± 1.34	3.80 ± 0.65	4.32 ± 7.78
*t*_1/2_ (h)	6.24 ± 1.53	9.80 ± 2.33	7.53 ± 1.49	7.51 ± 2.01
*T*_max_ (h)	1.83 ± 1.17	1.17 ± 0.68	3.76 ± 0.34	4.02 ± 1.33
CL/F (L/h/kg)	2.42 ± 0.40	1.46 ± 0.26	24.20 ± 5.46	21.35 ± 5.05
C_max_ (μg/L)	1767.67 ± 68.86	2309.67 ± 68.06	266.57 ± 35.56	286.74 ± 27.23

### Effect of anemarsaponin BII on the transport of nobiletin in Caco-2 cell model

**Figure 2.**
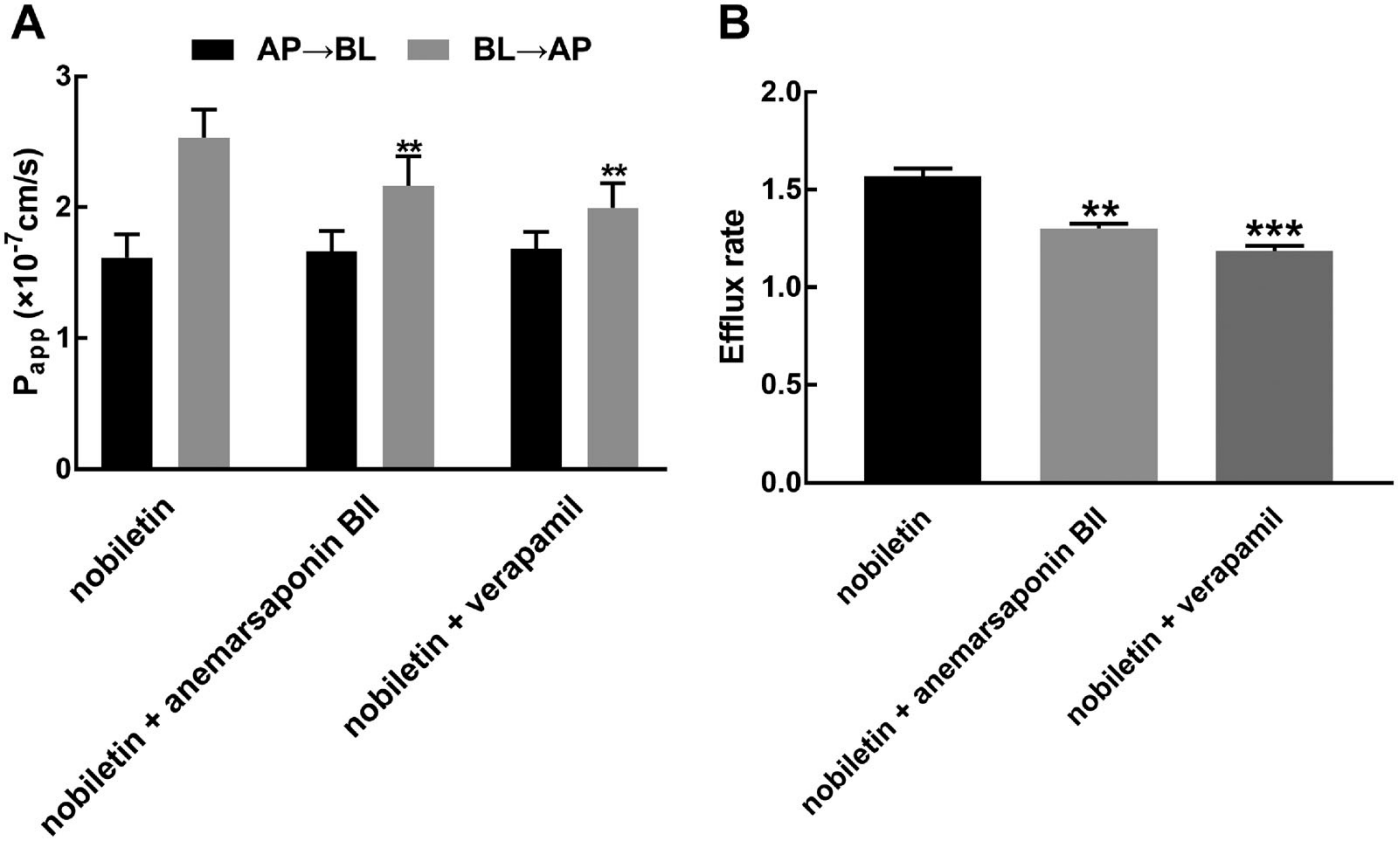
Effect of anemarsaponin BII on the transport of nobiletin. (A) Anemarsaponin BII and verapamil significantly suppressed the P_appBA_ value of nobiletin. (B) Anemarsaponin BII and verapamil showed a significant inhibitory effect on the efflux rate of nobiletin. **p < 0.01, ***p < 0.001 relative to the nobiletin alone group.

Verapamil significantly enhanced the value of P_appAB_ from 1.62 ± 0.18 × 10^−7 ^cm/s to 1.68 ± 0.13 × 10^−7 ^cm/s and suppressed the value of P_appBA_ from 2.53 ± 0.22 × 10^−7 ^cm/s to 1.19 ± 0.03 × 10^−7 ^cm/s (*p* < 0.01, Figure 2(A)). Meanwhile, the efflux rate of nobiletin was inhibited by verapamil from 1.57 ± 0.04 to 1.19 ± 0.03, indicating the involvement of *P-gp* in the transport of nobiletin (*p* < 0.001, Figure 2(B)). In the presence of anemarsaponin BII, the value of P_appAB_ increased to 1.66 ± 0.155 × 10^−7 ^cm/s and the value of P_appBA_ decreased to 2.17 ± 0.22 × 10^−7 ^cm/s (*p* < 0.01, Figure 2(A)). Correspondingly, the efflux rate of nobiletin also decreased to 1.30 ± 0.02 in the presence of anemarsaponin BII (*p* < 0.01, Figure 2(B)).

### Effect of anemarsaponin BII on the metabolic stability of nobiletin in rat liver microsomes

The half-life of nobiletin in rat liver microsomes was obtained as 31.56 min with the intrinsic clearance rate of 43.92 μL/min/mg protein. In the presence of anemarsaponin BII, the half-life of nobiletin is prolonged to 39.44 min with the intrinsic clearance rate decreasing to 35.15 μL/min/mg protein, suggesting the increasing metabolic stability of nobiletin and the potential involvement of CYP3A4 in the interaction between nobiletin and anemarsaponin BII during their co-administration.

### Effect of anemarsaponin BII on the activity of CYP3A4

**Figure 3.**
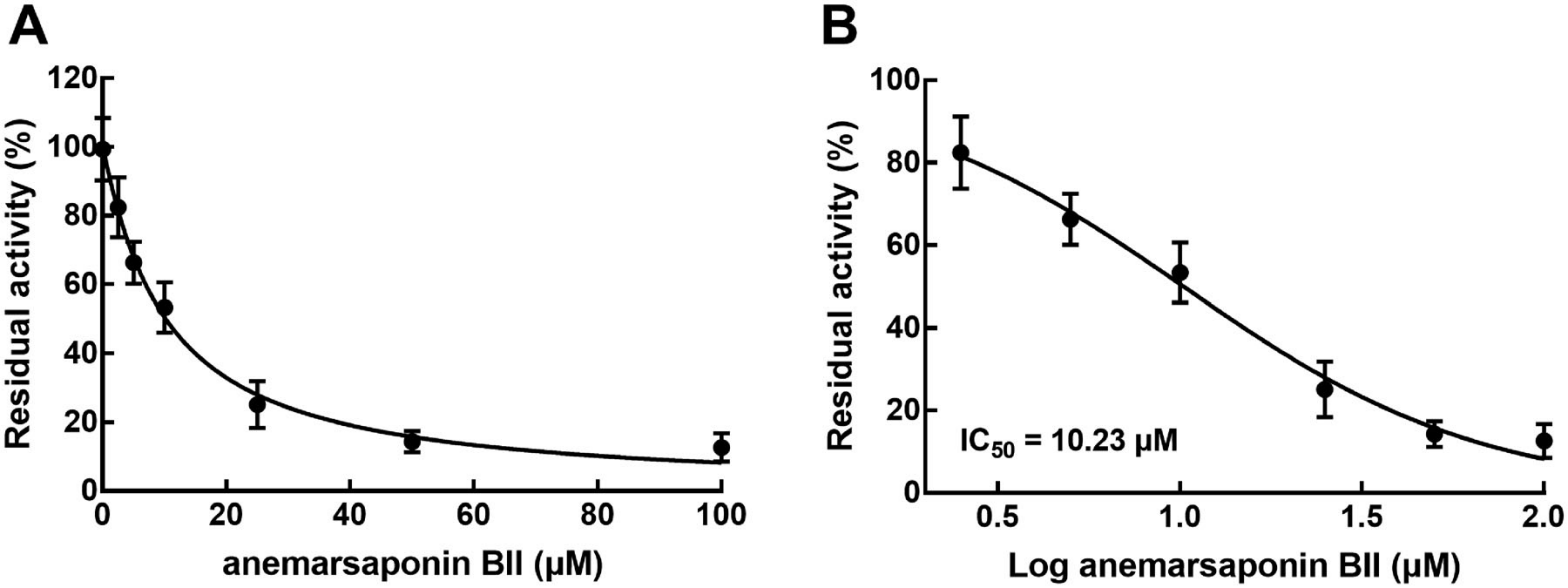
Effect of anemarsaponin BII on the activity of CYP3A4. (A) The activity of CYP3A4 decreased with the increasing concentration of anemarsaponin BII. (B) The IC_50_ value of anemarsaponin BII on CYP3A4 was obtained as 10.23 μM.

The involvement of CYP3A4 during the interaction of nobiletin with anemarsaponin BII was evaluated with rat liver microsomes. It was found that the activity of CYP3A4 decreased with the increasing concentration of anemarsaponin BII (Figure 3(A)), and the IC_50_ of anemarsaponin BII on CYP3A4 was obtained as 10.23 μM (Figure 3(B)).

## Discussion

Co-administration of different drugs could improve the therapeutic efficiency and benefit the treatment of comprehensive diseases (Falade et al. 2016). In traditional Chinese medicine, it is common to include herbs that possess similar or complementary pharmacological activity in one prescription. The interaction between these co-administrated herbs is critical to guide the clinical combination of different herbs. The interaction between active ingredients of different herbs that are responsible for the herb’s pharmacological activity has become research hotspots in recent studies, which can declaim the herb-herb interaction mechanically (Gong et al. 2015; Tao et al. 2019). Nobiletin and anemarsaponin BII are two major extractions of commonly used herbs in paediatrics, *C. reticulata*. and *A. asphodeloides* (Nogata et al. 2006; Sun et al. 2012). The interaction between these two extractions would affect the clinical sequel of their origin herbs.

The co-administration of nobiletin and anemarsaponin BII was investigated in this study. Anemarsaponin BII significantly changed the pharmacokinetic profile of nobiletin, indicating the interaction between nobiletin and anemarsaponin BII occurred. Consistently, anemarsaponin BII was found to improve the metabolic stability of nobiletin in rat liver microsomes reflected in the increased half-life and the decreasing intrinsic rate. Moreover, the liver is also the main metabolic organ that contains numerous cytochrome P450 enzymes (CYP450s) (Ingelman-Sundberg 2004). In previous studies, the activity of CYP450s has been considered as the major reason responsible for the drug-drug interaction (Gougis et al. 2021; Shibata et al. 2021; Zhou et al. 2021). CYP3A4 is the main enzyme involved in the metabolism of nobiletin (Takanaga et al. 2000; Weiss et al. 2020), and anemarsaponin BII was also revealed to inhibit the activity of CYP3A4 in a dose-dependent manner with the IC_50_ value of 10.23 μM in the present study, which is consistent with the previous study (Wang M et al. 2020). Hence, the inhibition of CYP3A4 was speculated to be one of the potential mechanisms underlying the interaction of nobiletin with anemarsaponin BII.

Additionally, *in vitro* experiments demonstrated that anemarsaponin BII also affected the transport of nobiletin. *P-gp* was demonstrated to participate in the transport of nobiletin, and the presence of anemarsaponin BII showed a dramatically inhibitory effect on the efflux of nobiletin. Therefore, the inhibition of *P-gp* by anemarsaponin BII was also considered as a potential mechanism during the interaction between nobiletin and anemarsaponin BII (Takanaga et al. 2000). However, the direct evidence of the *P-gp* inhibition by anemarsaponin BII lacked in the present study. Further investigations focussed the interaction between anemarsaponin BII and *P-gp* are needed in future studies.

## Conclusions

The co-administration of nobiletin and anemarsaponin BII induced adverse interaction that increased the system exposure and plasma concentration of nobiletin. Anemarsaponin BII was found to inhibit the activity of CYP3A4 and *P-gp*, which was speculated to be the potential mechanism underlying the herb-herb interaction between nobiletin and anemarsaponin BII. These results provide the theoretical basis for the clinical combination of *C. reticulata* and *A. asphodeloides*, and the dose of *C. reticulata*. should be adjusted when co-administrated with *A. asphodeloides*.
